# Differential response to anakinra and adalimumab in a patient with DADA2 syndrome

**DOI:** 10.1186/1546-0096-13-S1-P201

**Published:** 2015-09-28

**Authors:** B Toz, B Erer, S Kamali, L Ocal, A Gul

**Affiliations:** 1 Istanbul University, Istanbul Faculty of Medicine, Department of Internal Medicine, Istanbul, Turkey

## 

Deficiency of adenosine deiminase 2 (DADA2) syndrome is a recently described autosomal recessively inherited autoinflammatory disorder associated with missense mutations in CECR1 gene. Clinical manifestations include early onset stroke, livedoid vascular changes, and a vasculopathy mimicking classical polyarteritis nodosa (cPAN) characterized by microaneurysms and associated inflammatory findings. We herein describe a male patient with homozygous G47R mutation in the CECR1 gene, whose inflammatory findings did not respond to immunosuppressive treatments and recombinant IL-1 receptor antagonist, anakinra injections, but controlled with adalimumab.

## Case report

A 20-year-old male patient presented to our clinic two years ago with findings of cPAN. He had a consanguineous family from South East Turkey without any other affected individuals. He started to experience a skin rash at the age of 5, and he had a history of polypectomy following a rectal bleeding and then an explorative laparotomy due to skin rash, abdominal pain, and fever, with the diagnosis of Henoch Schönlein purpura at the age of 7. He also had hepatosplenomegaly, and he continued to experience 2-3 day lasting abdominal pain, especially after cold exposure until the age of 16. A skin biopsy revealed livedoid vasculitis. He also had a history of facial paralysis 3 years ago, and a sudden vision loss due to right retinal artery occlusion and hypertension 2 year ago. He was admitted to our hospital because of microaneurysms in cranial and renal arteries, with signs of renal infarcts as well as findings of mononeuritis multiplex and systemic inflammation. He was first diagnosed with familial Mediterranean fever (FMF) associated cPAN, and started to receive corticosteroids along with 3 courses of 500mg cyclophosphamide pulses. He then continued to receive azathioprine. MEFV gene screening revealed no exon 10 variations compatible with FMF diagnosis, and after the description of DADA2 syndrome, he was screened for CECR1 mutations, which revealed homozygous G47R mutation. During follow-up, he experienced transient ischemic attacks, and his acute phase response could not be controlled with cortiocoteroids and 2mg/kg azathioprine. After addition of 100 mg/day anakinra to his treatment, his acute phase response partially reduced and clinical findings remained stable for nearly 3 months, and increased doses of 200 mg/day did not provide additional help. However, switching his treatment to adalimumab dramatically reduced his CRP and ESR levels to normal limits.

## Conclusion

The pathogenesis and inflammatory characteristics of CECR1 mutation associated DADA2 syndrome and its vascular findings have not been elucidated yet, and a favorable response of our patient with DADA2 to adalimumab but not to anakinra treatment may provide insights to its inflammatory mechanisms.

## Consent to publish

Written informated consent for publication of their clinical details was obtained from the patient/parent/guardian/relative of the patient.

